# Changed frontal pole gene expression suggest altered interplay between neurotransmitter, developmental, and inflammatory pathways in schizophrenia

**DOI:** 10.1038/s41537-018-0044-x

**Published:** 2018-02-20

**Authors:** Elizabeth Scarr, Madhara Udawela, Brian Dean

**Affiliations:** 1 Molecular Psychiatry Laboratory, Florey Institute for Neuroscience and Mental Health, Parkville, VIC 3052 Australia; 2 CRC for Mental Health, Carlton, VIC 3053 Australia; 3 Melbourne Veterinary School, Faculty of Veterinary and Agricultural Sciences, The University of Melbourne, Parkville, VIC 3010 Australia; 4 Research Centre for Mental Health, the Faculty of Health, Arts and Design, Swinburne University, Hawthorne, VIC 3122 Australia

## Abstract

Schizophrenia (Sz) probably occurs after genetically susceptible individuals encounter a deleterious environmental factor that triggers epigenetic mechanisms to change CNS gene expression. To determine if omnibus changes in CNS gene expression are present in Sz, we compared mRNA levels in the frontal pole (Brodmann’s area (BA) 10), the dorsolateral prefrontal cortex (BA 9) and cingulate cortex (BA 33) from 15 subjects with Sz and 15 controls using the Affymetrix™ Human Exon 1.0 ST Array. Differences in mRNA levels (±≥20%; *p* < 0.01) were identified (JMP Genomics 5.1) and used to predict pathways and gene x gene interactions that would be affected by the changes in gene expression using Ingenuity Pathway Analysis. There was significant variation in mRNA levels with diagnoses for 566 genes in BA 10, 65 genes in BA 9 and 40 genes in BA 33. In Sz, there was an over-representation of genes with changed expression involved in inflammation and development in BA 10, cell morphology in BA 9 and amino acid metabolism and small molecule biochemistry in BA 33. Using 94 genes with altered levels of expression in BA 10 from subjects with Sz, it was possible to construct an interactome of proven direct gene x gene interactions that was enriched for genes in inflammatory, developmental, oestrogen, serotonergic, cholinergic and NRG1 regulated pathways. Our data shows complex, regionally specific changes in cortical gene expression in Sz that are predicted to affect homeostasis between biochemical pathways already proposed to be important in the pathophysiology of the disorder.

## Pathogenesis: the implication of differentially-expressed genes in frontal brain regions

Anterior brain regions exhibit significant amounts of differentially-expressed genes which might cause dysfunction in schizophrenia. It’s thought that schizophrenia occurs when environmental factors trigger gene expression changes and downstream effects in the human brain, though this is not fully understood. An Australian research group led by Brian Dean, from the Florey Institute of Neuroscience and Mental Health, conducted a post-mortem human brain study in which they compared gene expression between 15 schizophrenia patients and 15 controls. They found 566 instances of altered gene expression in the most frontal part of the brain, Brodmann Area 10, and fewer changes in proximal regions. These are brain areas known to mediate schizophrenia-related traits and the changes in gene expression in these areas will affect a range of essential biological pathways. The group also found 97 differentially-expressed genes that have been shown to directly interact with each. This study paints a complex picture of the causes of schizophrenia but suggests modern technologies can help unravel these complexities.

## Introduction

Schizophrenia (Sz) is a psychiatric disorder defined by the presence of a constellation of symptoms that includes positive symptoms (e.g., hallucinations and delusions), negative symptoms (e.g., anhedonia and social withdrawal) and cognitive deficits.^1^ Sz affects approximately 1% of the population worldwide, affects males and females equally and does not vary between urban and rural settings.^2^ The cause(s) of Sz remains to be elucidated but, whilst the disorder is heritable, studies in monozygotic twins show concordance rates of approximately 50%;^3^ this level of concordance is not consistent with a wholly genetic disorder. Thus, it is increasingly accepted that the disorder occurs in individuals with a genetic predisposition who encounter deleterious environmental factors that trigger functional changes in the central nervous system (CNS).^4^ It is now understood that in disorders that result from gene and environment interactions, the impact of environment is predominantly through epigenetic mechanisms that act to change gene expression.^5^ Hence, in Sz, it would be expected that there would be changes in gene expression in regions of the CNS that are dysfunctional in subjects with the disorder. A dysfunctional cortex is recognised as making a major contribution to the pathophysiology of Sz,^6^ it would therefore seem likely that changes in cortical gene expression could be contributing to the pathophysiology of the disorder.

The hypothesis that there are changes in gene expression in the cortex of from subjects with Sz has been addressed by measuring levels of mRNA using high-throughput technologies. The first such study, using gene expression microarrays, reported changes in levels of mRNA in the dorsolateral prefrontal cortex (DLPFC) from subjects with Sz that would impact on presynaptic functioning.^7^ Subsequently, studies in a number of cortical regions from subjects with Sz have reported changes in levels of expression of genes involved in G-protein signalling,^8,9^ energy and metabolism,^10–12^ controlling gene expression,^13,14^ protein degradation,^13^ glutamate neurotransmission,^9,11,13^ GABA neurotransmission,^15^ synaptic plasticity,^16^ neuronal development,^11,16–18^ neurotransmission,^11,16,18^ signal transduction,^16^ myelination,^11,16,19^ apoptosis,^11^ cell signalling,^11^ glial functioning,^19^ apolipoproteins,^20^ synaptic function,^9,18,21^ inflammation and immunity^14^ and sphingolipid metabolism.^22^ These studies argue that there are complex changes in gene expression affecting many biological pathways in the cortex of subjects with Sz. Moreover, one study showed that changes in gene expression varied between discrete cortical regions in Sz.^23^ To further explore the potential impact of changes in gene expression in discrete regions of the cortex on biological pathways, we decided to measure levels of mRNA in three cortical regions from a single cohort of subjects with Sz and an equal number of age and sex matched subjects with no history of mental illness (controls).

The human cortex has many anatomically distinct regions^24^ that show varying levels of functional connectivity.^25^ However, certain regions can exert strong control of specific behaviours as exampled by the frontal lobe which uniquely contributes to solving diverse cognitive problems and difficulties in managing such problems.^26^ Notably, abnormalities in these processes have been reported in subjects with Sz.^27^ Moreover, specific behaviours are known to be strongly influenced by specific regions within the frontal lobe. For example, the frontal pole has significant influence on relational reasoning and self-reflection,^28^ the DLPFC in involved in controlling working memory^29^ and the anterior cingulate cortex is involved decision-making^30^; all these cortical controlled functions have been reported as altered in Sz.^31–33^ Hence, we posited there would be changes in gene expression in the frontal pole (Brodmann’s area (BA) 10), the DLPFC (BA 9) and the anterior cingulate cortex (BA 33) from subjects with Sz.

## Results

### Demographics and sample collection data

There were no significant differences in age, gender ratio, postmortem interval (PMI) or CNS pH between the diagnostic groups (Table 1).

**Table 1:** Demographic, CNS collection and antipsychotic drug data for cases from which tissue was obtained to study gene expression in Brodmann’s areas 9, 10 and 33

	Age (yr.)	pH	PMI (hr.)	Brain Weight (gms)	Sex	Suicide	DI (yr.)	Cause of death	FRAD	FRADD*
Controls	59.3	6.58	20.5	1280	F			Congestive cardiac failure		
	62.1	6.45	40	1340	F			Ischaemic heart disease		
	80.8	6.28	55	1415	F			Ischaemic heart disease		
	32.3	6.16	56		F			Coronary atheroma		
	56.1	5.88	24	1200	F			Pericardial tamponade		
	38.8	6.26	52		F			Pulmonary thromboembolism		
	71.0	6.11	59	1400	M			Ischaemic heart disease		
	75.9	6.01	53	1140	F			Multiple organ failure		
	75.3	6.19	69.4	1360	M			Myocardial infarction		
	55.2	6.69	30.5	1253	M			Coronary artery atherosclerosis		
	52.5	6.52	33.8	1400	M			Cardiomegaly		
	67.0	6.44	49.3	1190	F			Aneurysm		
	66.4	6.47	71.8	1531	M			Coronary artery atheroma		
	42.8	6.45	30.5	1500	M			Ischaemic heart disease		
Mean	60	6.32	46	1334						
SD	14.5	0.23	16.3	124						
Ratio					6/8					
Schizophrenia										
	72.4	6.48	58.5		F	N	37	Pneumonia	Chlorpromazine	25
	59.1	6.44	46	1300	F	N	44	Congestive cardiac failure	Off drug	
	61.0	6.46	37.5	1365	M	N	38	Ischaemic heart disease	Off drug	
	69.1	6.44	48	1656	M	Y	6	Carbon monoxide poisoning	Off drug	
	38.0	6.43	20	1388	F	Y	17	Burning	Fluphenazine	485
	47.3	6.31	50	1570	F	N	20	Pneumonia	Off drug	
	30.0	6.37	48	1455	F	Y	10	Hanging	Chlorpromazine	600
	35.3	6.38	39	1480	F	Y	9	Incised wrists	Risperidone	150
	59.5	6.19	44.5	1110	F	N	35	Respiratory failure	Clozapine	632
	61.2	6.01	45.5	1256	M	Y	18	Hypovolaemic shock	Risperdone	285
	65.1	6.41	56	1124	M	N	46	Pneumonia with empyema	Olanzapine	270
	58.7	6.63	42.5	1240	M	N	36	Ischaemic heart disease	Zuclopentixol	506
	67.0	6.19	43.5	1344	M	N	30	Ischaemic heart disease	Off drug	
	82.3	6.13	46.5	1241	F	N	41	Cardiomegaly	Haloperidol prochlorperazine	216
	35.7	6.48	66	1659	M	Y	12	Overdose	Off drug	
Mean	56	6.36	46	1371			27			352
SD	15.4	0.16	10.4	177			13.9			211
*p*	0.53	0.63	0.99	0.55						
Ratios					7/8	6/9				
*p*					0.84					

*DI* duration of illness, *F* female, *FRAD* final recorded antipsychotic drug dose, FRADD* final recorded antipsychotic

drug dose converted to chlorpromazine equivalents per day, *M* male, *PMI* postmortem interval, *SD* standard deviation

### Gene expression arrays

No samples failed to pass QC. Some samples that passed QC but were on the edge of acceptability were identified. The impact of including these samples in our data analyses was assessed by systematic removal from the cohorts analysed and were shown not to affect the outcome of our analyses.

**Fig. 1:**
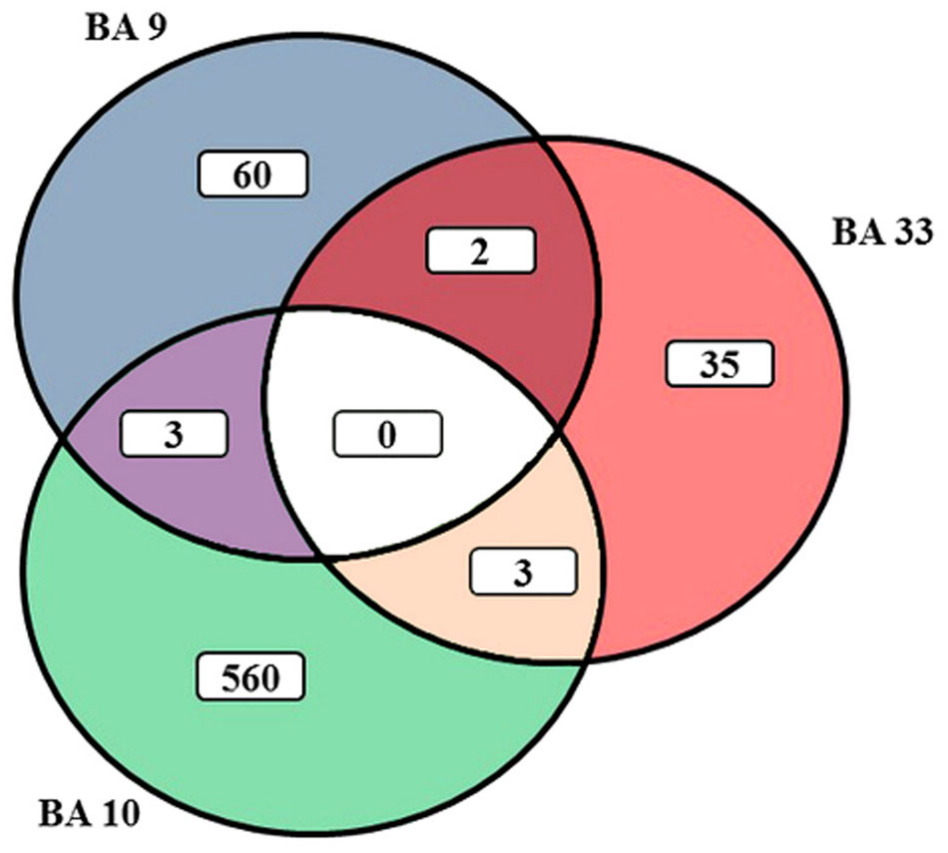
A Venn diagram showing the number of genes differentially expressed in Brodmann’s areas (BA) 9 (*n* = 60), 10 (*n* = 560) and 33 (*n* = 35) from subjects with schizophrenia compared to controls. The level of expression of no gene was significantly different in all three cortical regions from subjects with schizophrenia. Levels of expression of a limited number of genes were changed in more than one cortical region (BA 9 and BA 10 = 3, BA 9 and BA 33 = 2, BA 10 and BA 33 = 3)

Compared to controls, based on differential expression being ≥ 1.2-fold and *p* < 0.01, there were changes in gene expression in BA 9 (*n* = 65), BA 10 (*n* = 566) and BA 33 (*n* = 40) from subjects with Sz (Fig. 1). The expression of no gene was altered in all three cortical regions from subjects with Sz. Levels of expression of 2 genes (FUN14 domain containing 2 pseudogene 2 (*FUNDC2P2*) and MAGE family member A1 (*MAGEA1*)) were higher in BA 9 and BA 10 from subjects with the disorder. The expression of DnaJ heat shock protein family (Hsp40) member B4 (*DNAJB4*) was higher in BA 9 and lower in BA 10 from subjects with the disorder. Levels of expression of prune homolog 2 (*PRUNE2*) and solute carrier family 24 member 2 (*SLC24A2*) were lower in BA 9 and BA 33 from subjects with the disorder. The expression of actin, alpha 2, smooth muscle, aorta (*ACTA2*) was lower and bone morphogenetic protein 10 (*BMP10*) and microseminoprotein β (*MSMB*) higher in BA 10 and BA 33 from subjects with Sz.

In the study of the human genome, a number of approaches have been developed to control for false positive and false negative results that occur when analyzing large data sets.^34^ A number of approaches that attempt to control for false discovery rates, but not familywise error rates, are now used in the study of genomics and have been utilised in some transcriptomic studies.^35^ Whilst such an approach can control for the false discovery rate in studies using large cohorts they have a major flaw in causing a high number of true changes in gene expression being judged as false negatives.^34,35^ We used an FDR of 0.05, in a secondary analysis of our data, and no changes in gene expression survived because fold changes of >±1.98 with a *p* < 0.0000003 were needed. Given our previous experience using our differentiating criteria in being able to validate changes in gene expression using qPCR,^36,37^ we interpreted the results from our FDR analyses as reflecting the predicted overly high false negative rate associated with the use of FDR when diagnostic cohorts are less than 30 per cohort.

### Pathway analysis

IPA core analyses, using the Analysis Ready Genes (genes with changed levels of expression), was used to give insight into the potential impact of changes in gene expression on pathways and networks^38^ in the cortex of subjects with Sz. These analyses were focused on changes in gene expression in pathways and networks of relevance to CNS as the overall analyses also highlighted pathways and networks not relevant to CNS. Thus, there was a significant over representation of genes in BA 9 (18 genes: *p* = 4.95E^−02^) from subjects with Sz which have been linked to the presence of a neurological disease. In BA 10 (29 genes: *p* = 2.36E^−02^) and BA 33 (7 genes: *p* = 4.19E^−02^) from subjects with Sz, there was a significant over representation of genes link to developmental disorders whilst in BA 10 there were changes in expression of genes involved in infectious disease (inflammation: 38 genes: *p* = 4.16E^−02^). At physiological level, there was a significant over representation of genes involved in immune responsiveness (BA 9 = 10 genes: *p* = 3.74E^−02^, BA 33 = 14 genes: *p* = 3.60E^−02^) and embryonic development (BA 9 = 7 genes: *p* = 2.51E^−02^, BA 10 = 66 genes: *p* = 4.18E^−02^).

Of interest is the potential for changes in gene expression to impact on molecular and cellular functions. In this respect it was notable that there was an over representation of genes involved in cell to cell signaling and interaction (BA 9 = 9 genes: *p* = 4.95E^−02^, BA 10 = 70 genes: *p* = 4.18E^−02^, BA 33 = 14 genes: *p* = 4.19E^−02^), cell death and survival (BA 9 = 15 genes: *p* = 4.90E^−02^, BA 10 = 54 genes: *p* = 4.18E^−02^), cell cycle (BA 9 = 6 genes: *p* = 3.74E^−02^, BA 10 = 36 genes: *p* = 3.28E^−02^) and molecular transport (BA 10 = 37 genes: *p* = 4.18E^−02^, BA 33 = 14 genes: 4.19E^−02^) in the cortices from subjects with Sz.

### Molecular interactomes

**Fig. 2:**
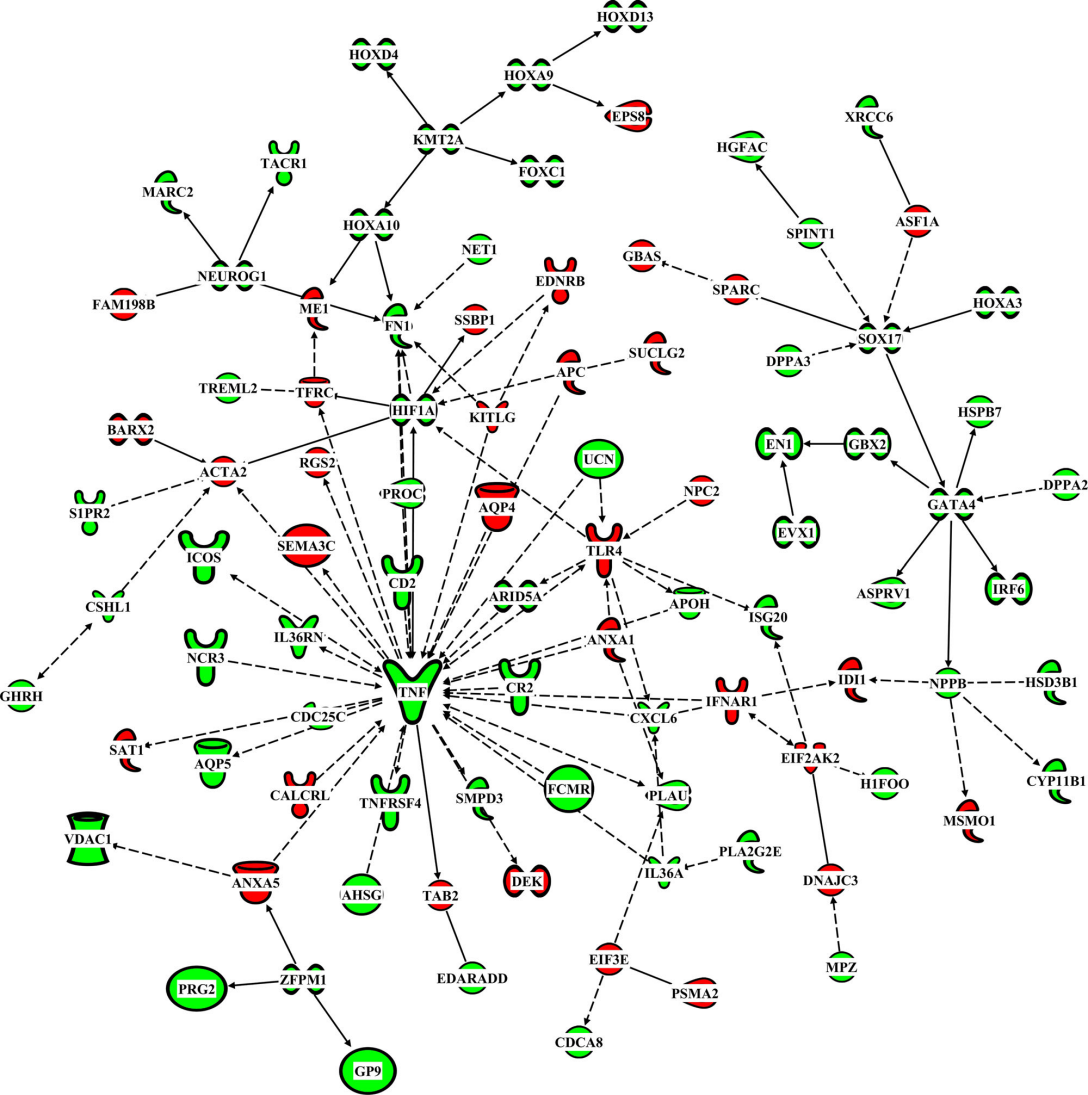
The interactions between 97 genes in Brodmann’s area 10 that had significant differences in levels of mRNA from subjects with schizophrenia compared to controls. Interactions only include direct gene x gene interactions that are experimentally proven using mammalian tissue. Colours indicate increased (green) or decreased (red) level of mRNA

IPA Pathway Explorer identifies known interactions between genes which allows the construction of gene interactomes. Our in silico analyses of known interactions between our Analysis Ready Genes in BA 10 from subjects with Sz compared to controls resulted in the construction of an interactome that included 94 of the 566 genes with altered levels of expression in that cortical region (Fig. 2). Interactomes including more than 2 gene could not be constructed from the 65 genes with altered levels of expression in BA 9 or the 40 genes with altered levels of expression in BA 33. The interactome of genes in BA 10 included 64 genes that had higher levels of expression in subjects with Sz (Fig. 2; Supplementary Table 1).

The genes in the BA 10 interactome contained multiple function classes (Supplementary Table 1) including cytokines, enzymes, G-protein coupled receptors, growth factors, an ion channel, a kinase, peptidases, a phosphatase, transcription regulators and transporters. The two largest classes of genes were enzymes (24%; *n* = 23) and transcriptional regulators (20%; *n* = 19). The genes in the interactome reside on 22 chromosomes (chromosomes 1–21 and X) with the largest grouping of genes per chromosome being on chromosome 2 (15%) and chromosome 1 (14%). A Core Analysis using the 97 genes in the interactome as the Analysis Ready Genes showed a significant over representation of genes involved in inflammatory response (46 genes; *p* = 2.05E^−4^–2.80E^−9^), cell death and survival (47 genes, *p* = 2.19E^−4^–2.39E^−11^), cell to cell signalling and interactions (33 genes, 1.90E^−04^–6.86E^−11^), cellular development (50 genes, *p* = 2.21E^−04^–1.67E^−10^) and cellular growth and proliferation (49 genes, *p* = 2.21E^−04^–1.67E^−10^). Particularly relevant to CNS function was the finding that genes in the interactome which were relatively over-represented included those regulated by oestrogen (11 genes; *p *= 4.92E^−05^), neuregulin 1 (9 genes; *p* = 1.30E^−04^), 5-hydoxytryptamine (8 genes, *p* = 5.64E^−03^) and acetylcholine (5 genes, *p* = 7.28E^−03^).

## Discussion

A major finding of this study is that the no gene had altered levels of expression in all three regions of the cortex from subjects with Sz. This finding is similar to that in another study that reported levels of expression of only 1 gene was altered in all 4 cortical regions (BA 21, 32, 38 and 46) from subjects with Sz when compared to controls.^39^ These data emphasise that, at the level of gene expression, the molecular footprint of Sz is complex and not uniform across the cortex. These data also raise the possibility that the symptoms of Sz that are thought to result from the dysfunction of different cortical regions^31–33^ could be due to changes in gene expression specific to the cortical region thought to be central to the genesis of a symptom.

We recently published data reporting changes in gene expression in BA 9 from subjects with Sz,^40^ only 10 of the genes with altered levels of expression in BA 9 in our current study were detected in our previous study using tissue from that cortical region. Significantly, our earlier study was designed to determine if levels of gene expression differed between a sub-group of subjects with a marked deficit in Sz (muscarinic receptor deficit Sz (MRDS)) compared to those with Sz and an absence of such a deficit. The cohort of subjects with Sz in that study was over-enriched (50%) with subjects with MRDS as our data suggests MRDS makes up 25% of subjects with Sz.^41^ By contrast, by chance, 20% of the subjects in this study would be classified as MRDS. Given we were able to detect differences in gene expression in BA 9 from subjects with MRDS compared to those without the deficit^40^ the difference in results between this and our previous study could be because our prior study did not contain a “typical” representative group of subjects with Sz.

A significant finding from our study is that there were many more changes in gene expression in the frontal pole, compared to the dorsolateral prefrontal cortex or the cingulate cortex, from subjects with Sz. Our data therefore suggests that the frontal pole is an area of the cortex where changes in gene expression could be having major effects on CNS function. The frontal pole is critical in maintaining the cognitive flexibility that underpins human reasoning and planning abilities,^42^ two CNS functions that are impaired in individuals with Sz.^43,44^ Thus, it is possible the extensive changes in gene expression we report in the frontal pole from subjects with Sz are involved in changes in cortical functioning that contribute to the symptoms of the disorder.

It is argued that changes in molecular pathways leading to altered cellular function are important components of the pathophysiology of Sz.^45^ It is therefore significant that our data suggests that changes in gene expression in the frontal pole and the DLPFC could be impacting on molecular and cellular functions involved in cell death and survival. This finding has relevance to findings in monozygotic twins discordant for Sz because many of the variations in nucleotide sequence between the twins were in genes involved in cell death and survival.^46^ Moreover, using cultured fibroblasts, it has been reported that cells from subjects who were drug naïve and in their first-episode of Sz had an increased susceptibility to apoptosis.^47^ This latter finding suggests changes in the activity of pathways involved in cell death and survival in subjects with Sz may extend beyond the CNS, are not simply due to effects of antipsychotic drug treatment and are not associated with a prolonged duration of illness. Finally, evidence at the level of proteomics also suggests changes in proteins in pathways involved in cell death are important in the pathophysiology of Sz.^48^ Notably, there is no data to suggest there are markedly increased levels of necrosis associated with apoptosis in the cortex of subjects with Sz.^49^ Hence, current data may be indicating there is an increase in cellular stress^50^ in the frontal pole and DLPFC, that may not extend to the anterior cingulate cortex, in subjects with Sz.

A third significant finding from our study, based on the existence of strong experimental evidence using mammalian tissue, is that interactions between 97 differentially expressed genes in the frontal pole could be contributing to changes in functioning of that CNS region. Significantly, there is prior evidence at the level of the gene, mRNA or protein that would support the proposition that 44 of the 97 genes in the frontal pole interactome are involved in the pathophysiology of Sz (Supplementary Table 2). Thus, a considerable body of research suggests that the genes and pathways affected by changes in gene expression in the BA 10 interactome could be contributing to the aetiology of the disorder. Significantly, tumour necrosis factor α (TNF) appears to be a critical gene in the BA 10 interactome which is significant as many studies suggest a role for this gene in the pathophysiology of Sz^51^ but it is only one of 27 genes with changed levels of expression that are known to be important in inflammatory related pathways. Moreover, 50 of the genes in the BA 10 interactome are known to be involved in developmental processes. Hence, the BA 10 interactome we highlight could be the first indication that a failure in interactions between neurodevelopmental and neuroinflammatory pathways could significantly contribute to the abnormal functioning of the frontal lobe in Sz. In addition, other major pathways that are predicted to be affected by such an integrated change in gene expression in the frontal pole from subjects with Sz are those regulated by oestrogen, serotonin, acetylcholine and neuregulin 1 and there is extensive evidence to implicate these pathways are involved in the pathophysiology of the disorder.^52–55^ In addition, oestrogen,^56,57^ serotonin,^58^ acetylcholine^59,60^ and neuregulin 1^61^ are known to control both developmental and inflammatory pathways, two overarching pathways likely to be affected by changes in expression of genes in the frontal pole interactome.

Like all studies on non-medication naïve subjects with Sz the outcomes of this study may reflect changes due to antipsychotic drug treatment, which would suggest the expression of our genes of interest are already targets for such drug treatments. Importantly, the distribution of the receptors targeted by antipsychotic drugs is relatively uniform across the frontal pole, DLPFC and anterior cingulate cortex; therefore, it would be unlikely antipsychotic drugs would have such differential effects on gene expression across 3 cortical regions. In addition, expression microarray studies have failed to show that levels of mRNA that we have shown to be altered Brodmann’s area 9, 10 or 33 from subjects with Sz are altered in rat cortex^62–64^ or striatum^65^ after treatment with a variety of antipsychotic drugs. Thus, available data supports the proposition that the changes in gene expression we report in Brodmann’s area 9, 10 and 33 from subjects with Sz are not likely to be simply due to antipsychotic drug treatment.

In conclusion, like a previous study,^39^ we have shown that different genes have altered levels of expression in different cortical regions from subjects with Sz, confirming Sz has a complex molecular pathophysiology. We also present evidence to support the proposition that 97 genes which have changed level of expression may be acting in concert to perturb the functioning of the frontal pole in subjects with Sz. Our findings may have some translational capability because TNFα,^66^ TLR4,^67^ ANXA1,^68^ HIF1A^69^ and PLAU,^70^ which are genes within the interactome, are already recognised as accessible drug targets. Therefore, a better understanding of changes in the biochemical pathways in the frontal pole interactome could be a precursor to the development of new treatments for Sz.

## Methods

### Human tissue collection and processing

Human CNS tissue was collected postmortem with approval from the Ethics Committee of the Victorian Institute of Forensic Medicine and the research was conducted in accordance with all relevant guidelines and procedures. All tissue was collected from cases coming to the Victorian Institute of Forensic Medicine after gaining written consent from the nearest next-of-kin. The cadavers from which tissue was collected were refrigerated within 5 h of being found. Importantly, the brain was removed at autopsy and the left hemisphere rapidly processed and frozen to −70 °C using a standardised procedure^71^ by the same individual in a way designed to minimise autolytic effects.^72^ The pH of the brain tissue was measured as described previously^73^ as this provides a good measure of overall tissue preservation.^74^ In addition, RNA integrity number (RIN) was measured as an indication of mRNA quality.^75^

The Human Ethics Committee of Melbourne Health gave permission to use CNS tissue for this study. All tissue was provided by the Victorian Brain Bank at the Florey Institute for Neuroscience and Mental Health. The tissue for these studies was taken from BA 9, BA 10 and BA 33 from the left CNS hemisphere of 15 subjects with Sz and 15 controls using classic cytoarchitectural markers. For BA 9, tissue was taken from the lateral surface of the frontal lobe from an area that includes the middle frontal gyrus superior to the inferior frontal sulcus. For BA 10, tissue was taken from the rostral portions of the superior frontal gyrus and the middle frontal gyrus that is bounded ventrally by the superior rostral sulcus but does not include the cingulate sulcus. For BA 33, tissue was taken from a narrow band located in the anterior cingulate gyrus that is adjacent to the supracallosal gyrus near the genu of the corpus callosum and that is bounded by the ventral anterior cingulate area 24 and the supracallosal gyrus. Importantly, previous experience has shown that diagnostic cohorts of this size gives meaningful results when analysed using the Affymetrix™ Human Exon 1.0 ST Arrays.^40^

### Case history review

Demographic, clinical and pharmacological and tissue collection data were obtained during a case history reviews conducted using the Diagnostic Instrument for Brain Studies (DIBS), as described previously.^76,77^ From the data collected using the DIBS, duration of illness was calculated as the time from first hospital admission to death, the final recorded dose of antipsychotic drugs (FRADD) was converted to a standardized drug dose (chlorpromazine equivalents per day; Chlor. Eqs.)^78^ and PMI was calculated as the time from death to autopsy. Where death was not witnessed, tissue was only collected from subjects who had been seen alive up to 5 h prior to being found dead, in these instances the PMI was taken as the midpoint between the person being found and being last seen alive. Diagnoses was made according to DSM-IV criteria.

### RNA preparation and microarrays processing

RNA preparation and expression array processing were as described previously.^40^ Essentially, total RNA was isolated from approximately 100 mg of frozen gray matter using 1.0 ml TRIzol^®^ reagent (Life Technologies, Australia) and, following homogenization and phase separation as per the manufacturer’s instructions, the aqueous phase was added to an equal volume of 70% ethanol. RNA was then isolated using RNeasy mini kits (Qiagen, Cat 74104). Hence, samples were treated with DNase using on column digestion with the absence of DNA contamination being confirmed using PCR and primers specific for the detection of the presence of genomic DNA. mRNA quantity and quality were analysed by spectrophotometry (NanoDrop; ThermoFisher Scientific) and by obtaining mRNA integrity numbers (RINs) using an Agilent 2100 bioanalyser (Agilent Technologies, CA, USA). All samples used for the microarray study had RINs > 7.00 (Range 7.30–9.20) as this is a good predictor of well-preserved RNA,^74^ which is required for acceptable microarray hybridization.

The Affymetrix™ Human Exon 1.0 ST Arrays (Affymetrix, Santa Clara, CA, USA), which were used to measure levels of mRNA in all cortical regions, were processed by the Ramaciotti Centre for Genomics at the University of New South Wales, where ribosomal mRNA was eliminated and random priming used to generate cRNA that was end labelled with biotin using the Affymetrix synthesis and labelling kit. Samples that passed the quality checkpoints were prepared for hybridisation using a standard probe cocktail. Each sample was loaded into an Affymetrix™ Human Exon 1.0 ST Array and hybridised overnight. Following post-hybridization washes, the chips were scanned and the fluorescent signals converted into a DAT file. After visual confirmation of the scans and quality control analysis, these files were used to generate subsequent cell intensity (CEL) and chip files for analysis. Data files are currently held by the CRC for Mental Health and will be made available to bona fide researchers upon request.

### Microarray data analyses

All CEL files were imported into JMP Genomics 5.1 (SAS, Cary, NS, USA) at the gene level, collapsing the exon level data onto known transcripts. To control for array to array variation, the data was normalized using the Robust Multichip Average algorithm. The data was then log2 transformed and because we planned to analyze across multiple datasets (diagnosis and region), normalized for between group comparisons using the “Least Square means” method.^79^ All QCs from each array were assessed using a distribution analysis to identify alterations in pattern expression followed by a one-way ANOVA to quantify any variation. A Correlation and Principal Variance Component Analysis was also run on the QC data, including Mahalanobis Distances analysis to identify any outliers. Using data that passed QC, JMP genomics was used to compare data from the subjects with Sz and the controls within each cortical region using an ANOVA (equivalent to t-test). To further reduce the likelihood of the inclusion of false positive results, we then follow the recognised standard practice of using a non-stringent group-wise *p*-value cutoff of <0.01 with a fold change of ±Δ 0.2^80^ as the final parameters used to filter genes with changed levels of expression in subjects with Sz. Using this approach, and two different Affymetrix arrays, we have previously shown that changes in gene expression reported identified using expression microarrays and postmortem CNS from subjects with Sz and age/sex matched controls are readily validated using quantitative polymerase chain reaction both within^14,36,37,40^ and outside^36,37,40^ of the cortical region in which expression array experiments were carried out and could be shown to have some diagnostic specificity.^36,37^ The data from these two studies suggests the rate of true positive differentially expressed genes is close to 90%.

### Gene expression pathway analyses

Genes with changed levels of expression in each of the three cortical regions from subjects with Sz (Analysis Ready Genes) were uploaded into Ingenuity Pathway Analysis build version 366632 M (IPA: http://www.ingenuity.com/products/ipa). Core analyses were completed using proven interactions in either direction, from data in the Ingenuity Knowledge Base (Genes Only) only using direct relationships that have been experimentally observed in human, rat or mouse tissue. The Core Analyses of our Analysis Ready Genes in IPA was completed by allocating genes automatically into classifications according to their known functions. Fisher’s right tailed exact test and the –log(*p*-value) was then used to identify pathways where there was a significant over representation of Analysis Ready Genes beyond what would be expected by chance by randomly allocating the total number of Analysis Ready Genes uploaded. The output from these analyses also include the number of genes with different levels of expression in the cortex of subjects with Sz as a ratio of the total number of genes included in any IPA classification by IPA and, where appropriate, the −log(Fisher’s Exact test) of the hypergeometric distribution of the data, which is used to rank networks according to their relevance to the dataset. Here we quote the most conservative estimates of the hypergeometric distribution p value.

In addition, using IPA, established relationships between genes with altered expression in the cortex from subjects with Sz were identified using Pathway Explorer. To attempt to exclude weak interactions settings were Direction: any, Data Sources: Ingenuity Expert Information only, Confidence Level: experimental observed and Species: human, mouse and rat using a Stringent Filter. No interactions that involved intermediary genes were included. Mutations were included and all types of relationships allowed. In limiting information used to the Ingenuity Expert Information any interactions will be those published in high-ranking journals after manual curation of the full text to establish contextual details.

### Data availability

The CEL files that support the findings of this study are held by the Cooperative Research Centre for Mental Health (http://www.mentalhealthcrc.com/) and are not currently publicly available as they contain data of commercial significance. However, the.CEL files can be made available following a confidentiality agreement being completed with the CRC for Mental Health. It is expected the.CEL files will become publicly available before the end of 2018.

## Electronic supplementary material

Supplementary Table 1

Supplementary Table 2
