# Supplementary material for: Changed frontal pole gene expression suggest altered interplay between neurotransmitter, developmental, and inflammatory pathways in schizophrenia
Source: NPJ Schizophr. 2018 Feb 20;4:4. doi: 10.1038/s41537-018-0044-x (PMC5820249; doi:10.1038/s41537-018-0044-x)
Supplement: Supplementary file 1 — Supplementary Table 1 [file 41537_2018_44_MOESM1_ESM.docx]

Supplementary Table 1: The functional group, physiological classification, gene name, chromosomal location, exon count, fold change in schizophrenia as a ratio of controls and significance of change for the genes in BA 10 included in the interactome constructed from changes in mRNA from subjects with schizophrenia.

| Function | Classification | Gene Name | Official Symbol | Chromosome | Exon Count | Fold Change | p |
| --- | --- | --- | --- | --- | --- | --- | --- |
| Development | Enzyme | APC, WNT signaling pathway regulator | APC | 5q22.2 | 18 | 0.77 | 0.0002 |
|  |  | aspartic peptidase retroviral like 1 | ASPRV1 | 2p13.3 | 1 | 1.21 | 0.0036 |
|  |  | cell division cycle 25C | CDC25C | 5q31.2 | 15 | 1.25 | 0.0097 |
|  |  |  |  |  |  |  |  |
|  | G-Couple Receptor | endothelin receptor type B | EDNRB | 13q22.3 | 9 | 0.68 | 0.0069 |
|  |  | regulator of G protein signaling 2 | RGS2 | 1q31.2 | 5 | 0.79 | 0.0064 |
|  |  | sphingosine-1-phosphate receptor 2 | S1PR2 | 19p13.2 | 2 | 1.29 | 0.0049 |
|  |  |  |  |  |  |  |  |
|  | Growth Factor | chorionic somatomammotropin hormone like 1 | CSHL1 | 17q23.3 | 5 | 1.21 | 0.0073 |
|  |  | KIT ligand | KITLG | 12q21.32 | 10 | 0.77 | 0.0018 |
|  |  |  |  |  |  |  |  |
|  | Other | alpha 2-HS glycoprotein | AHSG | 3q27.3 | 7 | 1.24 | 0.0005 |
|  |  | cell division cycle associated 8 | CDCA8 | 1p34.3 | 11 | 1.28 | 0.0069 |
|  |  | developmental pluripotency associated 2 | DPPA2 | 3q13.13 | 11 | 1.25 | 0.0030 |
|  |  | EDAR associated death domain | EDARADD | 1q42.3-q43 | 7 | 1.23 | 0.0019 |
|  |  | eukaryotic translation initiation factor 3 subunit E | EIF3E | 8q23.1 | 13 | 0.68 | 0.0022 |
|  |  | growth hormone releasing hormone | GHRH | 20q11.23 | 5 | 1.26 | 0.0019 |
|  |  |  |  |  |  |  |  |
|  | Transcription Regulator | AT-rich interaction domain 5A | ARID5A | 2q11.2 | 9 | 1.22 | 0.0092 |
|  |  | lysine methyltransferase 2A | KMT2A | 11q23.3 | 37 | 1.24 | 0.0045 |
|  |  | SRY-box 17 | SOX17 | 8q11.23 | 2 | 1.22 | 0.002 |
|  |  | developmental pluripotency associated 3 | DPPA3 | 12p13.31 | 4 | 1.53 | 0.0065 |
|  |  | engrailed homeobox 1 | EN1 | 2q14.2 | 2 | 1.23 | 0.0050 |
|  |  | forkhead box C1 | FOXC1 | 6p25.3 | 1 | 1.20 | 0.0062 |
|  |  | GATA binding protein 4 | GATA4 | 8p23.1 | 12 | 1.20 | 0.0016 |
|  |  | homeobox A3 | HOXA3 | 7p15.2 | 10 | 1.23 | 0.002 |
|  |  | homeobox A9 | HOXA9 | 7p15.2 | 2 | 1.24 | 0.0019 |
|  |  | homeobox A10 | HOXA10 | 7p15.2 | 3 | 1.26 | 0.0006 |
|  |  | homeobox D4 | HOXD4 | 2q31.1 | 2 | 1.23 | 0.0060 |
|  |  | homeobox D13 | HOXD13 | 2q31.1 | 4 | 1.31 | 0.0014 |
|  |  |  |  |  |  |  |  |
|  | Transmembrane Receptor | transferrin receptor | TFRC | 3q29 | 19 | 0.78 | 0.0031 |
|  |  |  |  |  |  |  |  |
|  |  |  |  |  |  |  |  |
| Inflammation / | Cytokine | C-X-C motif chemokine ligand 6 | CXCL6 | 4q13.3 | 4 | 1.23 | 0.0031 |
| Immune Response |  | interleukin 36 α | IL36A | 2q14.1 | 6 | 1.21 | 0.0065 |
|  |  | interleukin 36 receptor antagonist | IL36RN | 2q14.1 | 6 | 1.26 | 0.0077 |
|  |  | tumour necrosis factor | TNF | 6p21.33 | 4 | 1.28 | 0.0097 |
|  |  |  |  |  |  |  |  |
|  | Enzyme | annexin A1 | ANXA1 | 9q21.13 | 15 | 0.72 | 0.0086 |
|  |  | eukaryotic translation initiation factor 2 alpha kinase 2 | EIF2AK2 | 2p22.2 | 17 | 0.76 | 0.0096 |
|  |  | fibronectin 1 | FN1 | 2q35 | 47 | 1.25 | 0.0076 |
|  |  | interferon stimulated exonuclease gene 20 | ISG20 | 15q26.1 | 8 | 1.20 | 0.0018 |
|  |  | proteasome subunit alpha 2 | PSMA2 | 7p14.1 | 8 | 0.73 | 0.0018 |
|  |  | sphingomyelin phosphodiesterase 3 | SMPD3 | 16q22.1 | 15 | 1.21 | 0.0045 |
|  |  |  |  |  |  |  |  |
|  | Other | DnaJ heat shock protein family (Hsp40) member C3 | DNAJC3 | 13q32.1 | 16 | 0.75 | 0.0064 |
|  |  | Fc fragment of IgM receptor | FCMR | 1q32.1 | 8 | 1.24 | 0.0060 |
|  |  | TGF-beta activated kinase 1/MAP3K7 binding protein 2 | TAB2 | 6q25.1 | 15 | 0.80 | 0.0070 |
|  |  |  |  |  |  |  |  |
|  | Transcription Regulator | gastrulation brain homeobox 2 | GBX2 | 2q37.2 | 3 | 1.21 | 0.0047 |
|  |  | interferon regulatory factor 6 | IRF6 | 1q32.2 | 9 | 1.23 | 0.0033 |
|  |  |  |  |  |  |  |  |
|  | Transmembrane Receptor | CD2 molecule | CD2 | 1p13.1 | 5 | 1.26 | 0.0006 |
|  |  | complement C3d receptor 2 | CR2 | 1q32.2 | 21 | 1.20 | 0.0021 |
|  |  | inducible T-cell co-stimulator | ICOS | 2q33.2 | 5 | 1.27 | 0.0085 |
|  |  | interferon (alpha and beta) receptor 1 | IFNAR1 | 21q22.11 | 13 | 0.79 | 0.0081 |
|  |  | natural cytotoxicity triggering receptor 3 | NCR3 | 6p21.33 | 5 | 1.21 | 0.0027 |
|  |  | toll like receptor 4 | TLR4 | 9q33.1 | 4 | 0.76 | 0.0098 |
|  |  | TNF receptor superfamily member 4 | TNFRSF4 | 1p36.33 | 9 | 1.25 | 0.0036 |
|  |  |  |  |  |  |  |  |
|  | Transporter | annexin A5 | ANXA5 | 4q27 | 14 | 0.73 | 0.0031 |
|  |  |  |  |  |  |  |  |
|  |  |  |  |  |  |  |  |
| Transcription | Enzyme | anti-silencing function 1A histone chaperone | ASF1A | 6q22.31 | 4 | 0.71 | 0.0078 |
|  |  | epidermal growth factor receptor pathway substrate 8 | EPS8 | 12p12.3 | 22 | 0.75 | 0.0005 |
|  |  | X-ray repair cross complementing 6 | XRCC6 | 22q13.2 | 13 | 1.28 | 0.0092 |
|  |  |  |  |  |  |  |  |
|  | Other | H1 histone family member O oocyte specific | H1FOO | 3q22.1 | 6 | 1.21 | 0.0030 |
|  |  |  |  |  |  |  |  |
|  | Transcription Regulator | DEK proto-oncogene | DEK | 6p22.3 | 12 | 0.77 | 0.0047 |
|  |  | even-skipped homeobox 1 | EVX1 | 7p15.2 | 4 | 1.20 | 0.0089 |
|  |  |  |  |  |  |  |  |
| Cholesterol | Enzyme | cytochrome P450 family 11 subfamily B member 1 | CYP11B1 | 8q24.3 | 11 | 1.20 | 0.0071 |
|  |  | isopentenyl-diphosphate delta isomerase 1 | IDI1 | 10p15.3 | 7 | 0.74 | 0.0042 |
|  |  | malic enzyme 1 | ME1 | 6q14.2 | 14 | 0.80 | 0.0047 |
|  |  | methylsterol monooxygenase 1 | MSMO1 | 4q32.3 | 6 | 0.63 | 0.0013 |
|  |  |  |  |  |  |  |  |
|  | Other | NPC intracellular cholesterol transporter 2 | NPC2 | 14q24.3 | 4 | 0.76 | 0.0027 |
|  |  |  |  |  |  |  |  |
|  | Transporter | apolipoprotein H | APOH | 17q24.2 | 8 | 1.23 | 0.0046 |
|  |  |  |  |  |  |  |  |
|  | Other | natriuretic peptide B | NPPB | 1p36.22 | 3 | 1.23 | 0.0012 |
|  |  |  |  |  |  |  |  |
| Water Homeostasis | Transporter | aquaporin 4 | AQP4 | 18q11.2 | 6 | 0.69 | 0.0092 |
|  |  | aquaporin 5 | AQP5 | 12q13.12 | 5 | 1.20 | 0.0081 |
|  |  |  |  |  |  |  |  |
| Myelination | Structure | myelin protein zero | MPZ | 1q23.3 | 7 | 1.21 | 0.0065 |
|  |  |  |  |  |  |  |  |
| Other | Enzyme | hydroxy-delta-5-steroid dehydrogenase, 3 β and steroid δ-isomerase 1 | HSD3B1 | 1p12 | 4 | 1.22 | 0.0074 |
|  |  | hepatic growth factor activator | HGFAC | 4p16.3 | 15 | 1.22 | 0.0015 |
|  |  | mitochondrial amidoxime reducing component 2 | MARC2 | 1q41 | 11 | 1.22 | 0.0009 |
|  |  | phospholipase A2 group IIE | PLA2G2E | 1p36.13 | 4 | 1.21 | 0.0083 |
|  |  | plasminogen activator, urokinase | PLAU | 10q22.2 | 12 | 1.22 | 0.0040 |
|  |  | protein C, inactivator of coagulation factors Va and VIIIa | PROC | 2q14.3 | 9 | 1.20 | 0.0009 |
|  |  | spermidine/spermine N1-acetyltransferase 1 | SAT1 | Xp22.11 | 7 | 0.71 | 0.0071 |
|  |  | serine peptidase inhibitor, Kunitz type | SPINT1 | 15q15.1 | 11 | 1.21 | 0.0049 |
|  |  | succinate-CoA ligase GDP-forming beta subunit | SUCLG2 | 3p14.1 | 19 | 0.69 | 0.0072 |
|  |  |  |  |  |  |  |  |
|  | G-Couple Receptor | calcitonin receptor like receptor | CALCRL | 2q32.1 | 17 | 0.69 | 0.0003 |
|  |  | tachykinin receptor 1 | TACR1 | 2p12 | 5 | 1.23 | 0.0039 |
|  |  |  |  |  |  |  |  |
|  | Ion Channel | voltage dependent anion channel 1 | VDAC1 | 5q31.1 | 11 | 1.33 | 0.0025 |
|  |  |  |  |  |  |  |  |
|  | Other | actin, alpha 2, smooth muscle, aorta | ACTA2 | 10q23.31 | 10 | 0.77 | 0.0085 |
|  |  | family with sequence similarity 198 member B | FAM198B | 4q32.1 | 7 | 0.67 | 0.0046 |
|  |  | heat shock protein family B (small) member 7 | HSPB7 | 1p36.13 | 3 | 1.24 | 0.0005 |
|  |  | neuroepithelial cell transforming 1 | NET1 | 10p15.1 | 13 | 1.27 | 0.0020 |
|  |  | proteoglycan 2, pro eosinophil major basic protein | PRG2 | 11q12.1 | 6 | 1.23 | 0.0014 |
|  |  | semaphorin 3C | SEMA3C | 7q21.11 | 19 | 0.76 | 0.0018 |
|  |  | secreted protein acidic and cysteine | SPARC | 5q33.1 | 10 | 0.77 | 0.0018 |
|  |  | single stranded DNA binding protein 1 | SSBP1 | 7q34 | 9 | 0.78 | 0.0071 |
|  |  | triggering receptor expressed on myeloid cells like 2 | TREML2 | 6p21.1 | 5 | 1.26 | 0.0003 |
|  |  | urocortin | UCN | 2p23.3 | 2 | 1.32 | 0.0024 |
|  |  |  |  |  |  |  |  |
|  | Plasma Membrane | glioblastoma amplified sequence | GBAS | 19 | 10 | 0.73 | 0.0027 |
|  |  | glycoprotein IX platelet | GP9 | 3q21.3 | 6 | 1.24 | 0.0055 |
|  |  |  |  |  |  |  |  |
|  | Transcription Regulator | BARX homeobox 2 | BARX2 | 11q24.3 | 6 | 1.21 | 0.0085 |
|  |  | hypoxia inducible factor 1 alpha subunit | HIF1A | 14q23.2 | 16 | 0.77 | 0.0081 |
|  |  | neurogenin 1 | NEUROG1 | 5q31.1 | 1 | 1.20 | 0.0074 |
|  |  | zinc finger protein, FOG family member 1 | ZFPM1 | 16q24.2 | 14 | 1.21 | 0.0095 |
