# Supplementary material for: Changed frontal pole gene expression suggest altered interplay between neurotransmitter, developmental, and inflammatory pathways in schizophrenia
Source: NPJ Schizophr. 2018 Feb 20;4:4. doi: 10.1038/s41537-018-0044-x (PMC5820249; doi:10.1038/s41537-018-0044-x)
Supplement: Supplementary file 2 — Supplementary Table 2 [file 41537_2018_44_MOESM2_ESM.docx]

Supplementary Table 2: Genes with altered of expression in the BA 10 interactome for which there is prior evidence from the study of the genome, transcriptome or proteome which supports an involvement in the pathophysiology of schizophrenia.

| Gene Name | Official Symbol | Ref |
| --- | --- | --- |
| hydroxy-delta-5-steroid dehydrogenase, 3 beta- and steroid delta-isomerase 1 | HSD3B1 | ^1^ |
| CD2 molecule | CD2 | ^2^ |
| phospholipase A2 group IIE | PLA2G2E | ^3^ |
| heat shock protein family B (small) member 7 | HSPB7 | ^4^ |
| natriuretic peptide B | NPPB | ^5^ |
| TNF receptor superfamily member 4 | TNFRSF4 | ^6^ |
| myelin protein zero | MPZ | ^7^ |
| regulator of G protein signaling 2 | RGS2 | ^8^ |
| interferon regulatory factor 6 | IRF6 | ^9^ |
| complement C3d receptor 2 | CR2 | ^10^ |
| tachykinin receptor 1 | TACR1 | ^11^ |
| aspartic peptidase retroviral like 1 | ASPRV1 | ^12^ |
| eukaryotic translation initiation factor 2 alpha kinase 2 | EIF2AK2 | ^13^ |
| AT-rich interaction domain 5A | ARID5A | ^14^ |
| engrailed homeobox 1 | EN1 | ^15^ |
| protein C, inactivator of coagulation factors Va and VIIIa | PROC | ^16^ |
| inducible T-cell costimulator | ICOS | ^17^ |
| fibronectin 1 | FN1 | ^18^ |
| succinate-CoA ligase GDP-forming beta subunit | SUCLG2 | ^19^ |
| alpha 2-HS glycoprotein | AHSG | ^20^ |
| transferrin receptor | TFRC | ^21^ |
| annexin A5 | ANXA5 | ^22^ |
| APC, WNT signaling pathway regulator | APC | ^23^ |
| voltage dependent anion channel 1 | VDAC1 | ^22^ |
| neurogenin 1 | NEUROG1 | ^24^ |
| cell division cycle 25C | CDC25C | ^25^ |
| triggering receptor expressed on myeloid cells like 2 | TREML2 | ^26^ |
| tumor necrosis factor | TNF | ^27^ |
| natural cytotoxicity triggering receptor 3 | NCR3 | ^28^ |
| DEK proto-oncogene | DEK | ^29^ |
| proteasome subunit alpha 2 | PSMA2 | ^30^ |
| even-skipped homeobox 1 | EVX1 | ^31^ |
| semaphorin 3C | SEMA3C | ^32^ |
| single stranded DNA binding protein 1 | SSBP1 | ^22^ |
| eukaryotic translation initiation factor 3 subunit E | EIF3E | ^33^ |
| annexin A1 | ANXA1 | ^34^ |
| toll like receptor 4 | TLR4 | ^35^ |
| isopentenyl-diphosphate delta isomerase 1 | IDI1 | ^36^ |
| actin, alpha 2, smooth muscle, aorta | ACTA2 | ^36^ |
| KIT ligand | KITLG | ^37^ |
| hypoxia inducible factor 1 alpha subunit | HIF1A | ^38^ |
| zinc finger protein, FOG family member 1 | ZFPM1 | ^39^ |
| apolipoprotein H | APOH | ^40^ |
| aquaporin 4 | AQP4 | ^41^ |

References

1 Lee, Y. H., Kim, J.-H. & Song, G. G. Pathway analysis of a genome-wide association study in schizophrenia. *Gene* **525**, 107-115, doi:<https://doi.org/10.1016/j.gene.2013.04.014> (2013).

2 Kumarasinghe, N. *et al.* Gene expression profiling in treatment-naive schizophrenia patients identifies abnormalities in biological pathways involving AKT1 that are corrected by antipsychotic medication. *Int. J. Neuropsychopharmacol.*, 1-21 (2013).

3 Law, M. H., Cotton, R. G. & Berger, G. E. The role of phospholipases A2 in schizophrenia. *Mol Psychiatry* **11**, 547-556, doi:10.1038/sj.mp.4001819 (2006).

4 Kowalczyk, M. *et al.* Heat shock protein 70 gene polymorphisms are associated with paranoid schizophrenia in the Polish population. *Cell Stress Chaperones* **19**, 205-215, doi:10.1007/s12192-013-0446-7 (2014).

5 Politi, P., Minoretti, P., Piaggi, N., Brondino, N. & Emanuele, E. Elevated plasma N-terminal ProBNP levels in unmedicated patients with major depressive disorder. *Neurosci. Lett.* **417**, 322-325, doi:<https://doi.org/10.1016/j.neulet.2007.02.056> (2007).

6 Lane, J. M. *et al.* Genome-wide association analysis identifies novel loci for chronotype in 100,420 individuals from the UK Biobank. *Nat Commun* **7**, 10889, doi:10.1038/ncomms10889 (2016).

7 Uranova, N. A., Vostrikov, V. M., Orlovskaya, D. D. & Rachmanova, V. I. Oligodendroglial density in the prefrontal cortex in schizophrenia and mood disorders: a study from the Stanley Neuropathology Consortium. *Schizophr. Res.* **67**, 269-275 (2004).

8 Campbell, D. B. *et al.* Association of RGS2 and RGS5 variants with schizophrenia symptom severity. *Schizophr. Res.* **101**, 67-75, doi:10.1016/j.schres.2008.01.006 (2008).

9 Gurling, H. M. D. *et al.* Genomewide Genetic Linkage Analysis Confirms the Presence of Susceptibility Loci for Schizophrenia, on Chromosomes 1q32.2, 5q33.2, and 8p21-22 and Provides Support for Linkage to Schizophrenia, on Chromosomes 11q23.3-24 and 20q12.1-11.23. *Am. J. Hum. Genet.* **68**, 661-673 (2001).

10 Sanders, A. R. *et al.* Transcriptome sequencing study implicates immune-related genes differentially expressed in schizophrenia: new data and a meta-analysis. *Trans.Psychiatr.* **7**, e1093, doi:10.1038/tp.2017.47 (2017).

11 Tooney, P. A., Crawter, V. C. & Chahl, L. A. Increased tachykinin NK(1) receptor immunoreactivity in the prefrontal cortex in schizophrenia. *Biol. Psychiatr.* **49**, 523-527 (2001).

12 Viana, J. *et al.* Schizophrenia-associated methylomic variation: molecular signatures of disease and polygenic risk burden across multiple brain regions. *Hum. Mol. Genet.* **26**, 210-225, doi:10.1093/hmg/ddw373 (2017).

13 English, J. A. *et al.* Reduced protein synthesis in schizophrenia patient-derived olfactory cells. *Transl Psychiatry* **5**, e663, doi:10.1038/tp.2015.119 (2015).

14 Ayalew, M. *et al.* Convergent functional genomics of schizophrenia: from comprehensive understanding to genetic risk prediction. *Mol Psychiatry* **17**, 887-905, doi:10.1038/mp.2012.37 (2012).

15 Webb, B. T., Sullivan, P. F., Skelly, T. & van den Oord, E. J. Model-based gene selection shows engrailed 1 is associated with antipsychotic response. *Pharmacogenet. Genomics* **18**, 751-759, doi:10.1097/FPC.0b013e32830162bc (2008).

16 Ng, M. Y. M. *et al.* Meta-analysis of 32 genome-wide linkage studies of schizophrenia. *Mol. Psychiatr.* **14**, 774-785, doi:10.1038/mp.2008.135 (2009).

17 Tomkiewicz, A. M. *et al.* in *15th International Congress of Immunology* (Frontiers in Immunology, Milan, 2013).

18 Miyamae, Y. *et al.* Altered adhesion efficiency and fibronectin content in fibroblasts from schizophrenic patients. *Psychiatry Clin. Neurosci.* **52**, 345-352, doi:10.1046/j.1440-1819.1998.00386.x (1998).

19 Kim, J. *et al.* Somatic deletions implicated in functional diversity of brain cells of individuals with schizophrenia and unaffected controls. *Sci. Rep.* **4**, 3807, doi:10.1038/srep03807 (2014).

20 Levin, Y. *et al.* Global proteomic profiling reveals altered proteomic signature in schizophrenia serum. *Mol Psychiatry* **15**, 1088-1100, doi:10.1038/mp.2009.54 (2010).

21 Maes, M., Meltzer, H. Y., Buckley, P. & Bosmans, E. Plasma-soluble interleukin-2 and transferrin receptor in schizoprenia and major depression. *Eur. Arch. Psychiatry Clin. Neurosci.* **244**, 325-329, doi:10.1007/bf02190412 (1995).

22 Focking, M. *et al.* Proteomic and genomic evidence implicates the postsynaptic density in schizophrenia. *Mol Psychiatry* **20**, 424-432, doi:10.1038/mp.2014.63 (2015).

23 Cui, D. H., Jiang, K. D., Jiang, S. D., Xu, Y. F. & Yao, H. The tumor suppressor adenomatous polyposis coli gene is associated with susceptibility to schizophrenia. *Mol Psychiatry* **10**, 669-677 (2005).

24 Fanous, A. H. *et al.* Association between the 5q31.1 gene neurogenin1 and schizophrenia. *Am.J.Med.Genet. Part B: Neuropsychiatr. Genet.* **144B**, 207-214, doi:10.1002/ajmg.b.30423 (2007).

25 Schizophrenia Working Group of the Psychiatric Genomics, C. *et al.* Biological Insights From 108 Schizophrenia-Associated Genetic Loci. *Nature* **511**, 421-427, doi:10.1038/nature13595 (2014).

26 Yoshino, Y. *et al.* DNA Methylation Changes in Intron 1 of Triggering Receptor Expressed on Myeloid Cell 2 in Japanese Schizophrenia Subjects. *Front. Neurosci.* **11**, 275, doi:10.3389/fnins.2017.00275 (2017).

27 O'Brien, S. M., Scully, P. & Dinan, T. G. Increased tumor necrosis factor-alpha concentrations with interleukin-4 concentrations in exacerbations of schizophrenia. *Psychiatry Res.* **160**, 256-262 (2008).

28 Andreassen, O. A. *et al.* Genetic pleiotropy between multiple sclerosis and schizophrenia but not bipolar disorder: differential involvement of immune-related gene loci. *Mol Psychiatry* **20**, 207-214, doi:10.1038/mp.2013.195 (2015).

29 Vawter, M. P., Mamdani, F. & Macciardi, F. An integrative functional genomics approach for discovering biomarkers in schizophrenia. *Briefings in Functional Genomics* **10**, 387-399, doi:10.1093/bfgp/elr036 (2011).

30 Arion, D. *et al.* Distinctive transcriptome alterations of prefrontal pyramidal neurons in schizophrenia and schizoaffective disorder. *Mol Psychiatry* **20**, 1397-1405, doi:10.1038/mp.2014.171 (2015).

31 Liang, J. *et al.* Single-trait and multi-trait genome-wide association analyses identify novel loci for blood pressure in African-ancestry populations. *PLoS Genetics* **13**, e1006728, doi:10.1371/journal.pgen.1006728 (2017).

32 Arion, D., Horváth, S., Lewis, D. A. & Mirnics, K. Infragranular gene expression disturbances in the prefrontal cortex in schizophrenia: Signature of altered neural development? *Neurobiol. Dis.* **37**, 738-746, doi:<https://doi.org/10.1016/j.nbd.2009.12.013> (2010).

33 Mistry, M., Gillis, J. & Pavlidis, P. Genome-wide expression profiling of schizophrenia using a large combined cohort. *Mol Psychiatry* **18**, 215-225, doi:10.1038/mp.2011.172 (2013).

34 Clark, D., Dedova, I., Cordwell, S. & Matsumoto, I. A proteome analysis of the anterior cingulate cortex gray matter in schizophrenia. *Mol. Psychiatr.* **11**, 459-470 (2006).

35 García-Bueno, B. *et al.* Evidence of activation of the Toll-like receptor-4 proinflammatory pathway in patients with schizophrenia. *Journal of Psychiatry & Neuroscience : JPN* **41**, E46-E55, doi:10.1503/jpn.150195 (2016).

36 Prabakaran, S. *et al.* Mitochondrial dysfunction in schizophrenia: evidence for compromised brain metabolism and oxidative stress. *Mol. Psychiatr.* **9**, 684-697 (2004).

37 Dean, B. Dissecting the Syndrome of Schizophrenia: Progress toward Clinically Useful Biomarkers. *Schizophr.Res.Treatment.* **2011**, 614730 (2011).

38 Richards, A. L. *et al.* Schizophrenia susceptibility alleles are enriched for alleles that affect gene expression in adult human brain. *Mol. Psychiatr.* **17**, 193, doi:10.1038/mp.2011.11

<https://www.nature.com/articles/mp201111#supplementary-information> (2011).

39 Montano, C., Taub, M. A., Jaffe, A. & et al. Association of dna methylation differences with schizophrenia in an epigenome-wide association study. *JAMA psychiatry* **73**, 506-514, doi:10.1001/jamapsychiatry.2016.0144 (2016).

40 Chan, M. K. *et al.* Development of a blood-based molecular biomarker test for identification of schizophrenia before disease onset. *Transl Psychiatry* **5**, e601, doi:10.1038/tp.2015.91 (2015).

41 Muratake, T., Fukui, N., Kaneko, N., Amagane, H. & Someya, T. Linkage disequilibrium in aquaporin 4 gene and association study with schizophrenia. *Psychiatry Clin. Neurosci.* **59**, 595-598, doi:10.1111/j.1440-1819.2005.01420.x (2005).
